# Supplementary material for: Mutation, selection, and the prevalence of the Caenorhabditis elegans heat-sensitive mortal germline phenotype
Source: G3 (Bethesda). 2022 Mar 21;12(5):jkac063. doi: 10.1093/g3journal/jkac063 (PMC9073675; doi:10.1093/g3journal/jkac063)
Supplement: jkac063_Supplementary_Figure_S1 [file jkac063_supplementary_figure_s1.docx]

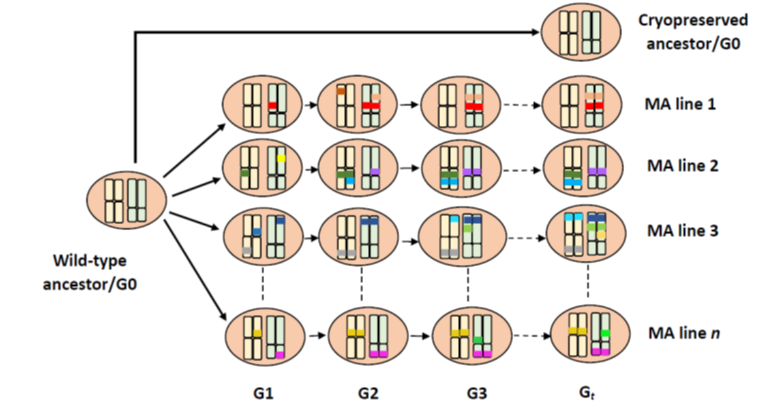


**Figure S1**. Schematic depiction of the mutation accumulation (MA) experiment. Yellow and green bars represent chromosomes; colored bars on the chromosomes represent mutations unique to a specific MA line. The ancestor (G0) was cryopreserved at the outset of the experiment. G_t_=250 generations. See Methods for details.
