# Supplementary material for: Mutation, selection, and the prevalence of the Caenorhabditis elegans heat-sensitive mortal germline phenotype
Source: G3 (Bethesda). 2022 Mar 21;12(5):jkac063. doi: 10.1093/g3journal/jkac063 (PMC9073675; doi:10.1093/g3journal/jkac063)
Supplement: jkac063_Supplementary_Figure_S2 [file jkac063_supplementary_figure_s2.pdf]

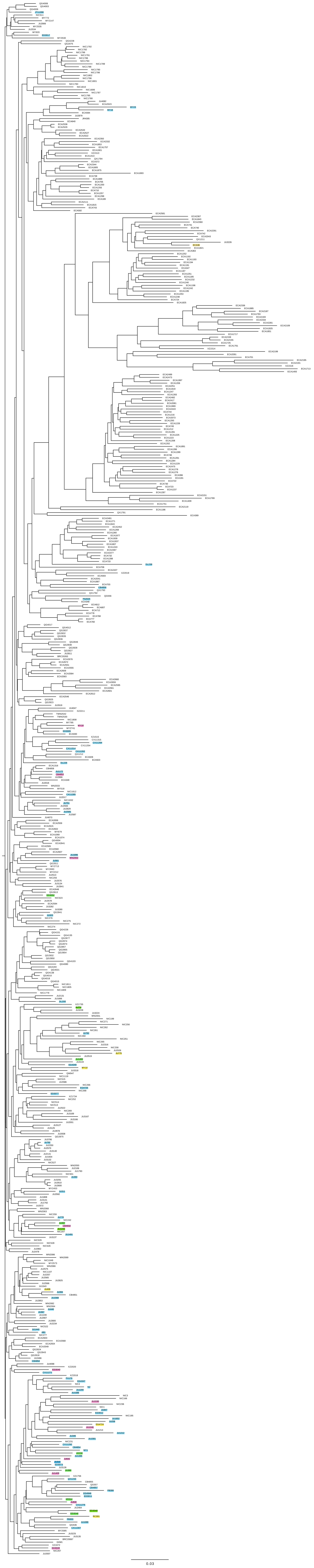
0.03

**Figure S2**. Neighbor-Joining tree of *C. elegans* whole-genome haplotypes. Strains highlighted in yellow are strong Mrt phenotype, green are moderate Mrt, purple are weak Mrt, and blue are wild-type. See Methods for details of phylogeny reconstruction and description of Mrt phenotypes.
